# Instructions in Manipulation of Hard Rubber or Vulcanite

**Published:** 1874-11

**Authors:** 


*Instructions in Manipulations of Hard Rubber or Vulcanite for Dental Purposes.* By E. Wildman, M. D., D. D. S., Professor of Mechanical Dentistry in the Penna. College of Dental Surgery. Sixth Edition. Publisher, Samuel S. White, Philadelphia, 1875.

The number of editions is proof of the value of this work, which is written in an easy and comprehensive style, and after Professor Austen's instructions on Plastic Work in the last edition of Harris Principles and Practice of Dentistry, forms the best treatise published. All that it is necessary to know concerning "Vulcanite Work" is contained in this volume, which is hansomely bound and printed in large type on good paper; and is altogether in keeping with the productions of its enterprising publisher.

